# Tinnitus Education for Audiologists Is a Ship at Sea: Is It Coming or Going?

**DOI:** 10.3390/audiolres13030034

**Published:** 2023-05-25

**Authors:** Marc Fagelson

**Affiliations:** 1 Department of Audiology and Speech Language Pathology, East Tennessee State University, Johnson City, TN 37614, USA; fagelson@etsu.edu; 2 James H. Quillen Mountain Home VAMC, Johnson City, TN 37684, USA

**Keywords:** tinnitus, cognitive behavioral therapy, self-efficacy, doctor of audiology

## Abstract

Subjective tinnitus is a highly prevalent sound sensation produced in most cases by persistent neural activity in the auditory pathway of the patient. Audiologists should be confident that they can employ elements of sound therapy and related counseling to support patients in coping. However, patients with bothersome tinnitus may be challenged by mental health complications, and they struggle to find adequate care when tinnitus and psychological distress co-occur. Audiologists in many cases lack the confidence to provide in-depth counseling while mental health providers lack basic understanding of tinnitus, its mechanisms, and the elements of audiologic management that could support patients in coping. At the very least, audiologists should be able to explain the mechanisms involved in and contributing to negative tinnitus effects, conduct valid measures of these effects, and offer reasonable options for managing the consequences linked by the patient to bothersome tinnitus and sound-related sensations. This brief communication summarizes the current state of tinnitus-related opportunities offered in US audiology training programs, and the substantial need to improve both the education of practitioners and the delivery of services to patients in need.

## 1. Introduction

Subjective tinnitus is not associated with sounds external to the body; however, its onset and detectability may be related to a specific medical condition, such as otosclerosis or otitis media [1], or it can arise from, and provide a reminder of, a specific event such as traumatic acoustic overexposure [2,3]. Tinnitus may also arise as a consequence of ototoxic medication use [4,5]. Subjective tinnitus may appear idiopathically, intermittently, and be unrelated to auditory thresholds [6,7]. Tinnitus often co-occurs with other auditory symptoms such as intolerance to moderate levels of sound (i.e., hyperacusis) or powerful negative emotions such as anger or rage in response to sounds that do not bother other people (i.e., selective sound sensitivity or misophonia) [8]. The diversity of these experiences, triggers, and consequences challenges providers as patients require counseling not only for audiologic rehabilitation, but often interventions for co-occurring mental health complications. 

The education of practitioners in this area of audiology is of obvious value, and although published standards [9] reference prevention and management, there remains substantial heterogeneity among audiology programs in the US with regard to classroom and clinical opportunities to study tinnitus and sound tolerance disorders. Reports concluding that psychological management of tinnitus may for many patients provide better outcomes than audiologic management can point both audiology and clinical psychology fields to reasonable interventions (i.e., cognitive behavioral therapy (CBT)) [10]. However, such findings may not encourage audiologists who lack experience and comfort with counseling regarding tinnitus to provide management services for distressed patients. Patients often express frustration and disappointment when seeking clinical services for tinnitus and it is not uncommon for a patient, particularly one who experiences co-occurring mental health disorders, to cease searching despite needing help [11].

## 2. Methods

This communication summarizes elements of audiology education that can be considered as viable avenues through which programs and students may improve current delivery of tinnitus-related clinical services. Academic and clinical programs routinely assess their resources as they face choices regarding the opportunities they provide. Few programs provide comprehensive and consistent opportunities for their students that meet the needs of patients who have bothersome tinnitus and lack access to dependable care. Current practices in clinical psychology that have relevance and value to audiologists, and that audiologists conduct routinely within their scope of practice, such as fitting hearing aids to reluctant patients and counseling parents of hearing-impaired children, are summarized below.

## 3. Results

### 3.1. Tinnitus in the Doctor of Audiology Program

Henry et al. [12] conducted a survey of 75 academic programs offering the AuD degree. The survey focused on class and clinic activity directly supporting tinnitus education and services. In total, 32 out of 75 programs responded to the survey. The results depicted a sub-optimal situation in which only a handful of programs (5) specified an hourly total related to tinnitus coursework, while 15/32 programs indicted less than one credit hour (or sixteen total hours of class and clinic time) devoted to tinnitus training. Only 10/32 programs indicated an hourly total commensurate with 1 full-semester course focused on tinnitus, including student clinic assignments that provided contact with patients reporting tinnitus. It may be an overly optimistic interpretation to consider the non-responder programs (i.e., approximately 1/3 of the remaining 43 programs) as offering a similar amount of tinnitus study and opportunities to students. 

Henry et al. [12] questioned programs regarding management approaches taught or offered in clinical activity. All but one program indicated instruction/experience related to masking/sound therapy, 91% specified instruction in tinnitus retraining therapy (TRT) [13] and in progressive tinnitus management (PTM) [14]. Other interventions such as tinnitus activities treatment (TAT) [15] and cognitive behavioral therapy (CBT) [16] were specified by programs less often; unfortunately, the Henry et al. [12] summary lacked intervention fidelity information. Therefore, while it was encouraging that at least the responding programs endeavored to support the management of patients with tinnitus, the degree to which their didactic and clinical instruction articulated information essential to the interventions was not assessed. Other interventions specified (pharmacological, counseling, mindfulness, and stress reduction) either required other professionals for implementation, or lacked specific guidelines commensurate with those published for TRT, PTM, CBT, or TAT. Two programs mentioned instruction regarding hearing aid use; however, hearing aid use likely would be implemented as well by those programs specifying masking/sound therapy. 

A recent European guideline [10] offered a comprehensive review of diagnostic, assessment, and treatment approaches related to tinnitus patients and providers. The authors identified barriers to and facilitators of tinnitus research and clinical activity, at one point indicating that fewer than 50% of respondents in countries that offered tinnitus services were satisfied with the care they received. Among the barriers, a lack of specialty clinics, a lack of time for counseling and assessment, difficulties related to payment for services, in addition to variability in treatment protocols could be generalized as challenges present concurrently in the US. With regard to facilitators of care, the authors specified among other items the condition’s ubiquity, and an awareness that services remain inadequate. 

Professional organizations serving audiologists, as well as academic programs and specialty continuing-education outlets, offer additional certification or training opportunities for clinicians. These programs are in most cases offered online, using asynchronous delivery, and employ modules related to different aspects of tinnitus mechanisms and management. Therefore, the challenges of coordinating clinical opportunities with classroom work might not be easily nor reasonably addressed. Ultimately, learners must translate the educational components into routine clinical practice, and while the online programs endeavor to address gaps in training, their effectiveness may be tempered by an unavoidable disconnect if face-to-face clinical opportunities are not provided concurrently. 

As the development and delivery of tinnitus programs remains a challenge, their sustainability must also be addressed. The American Speech-Language Hearing Association (ASHA) offered 591 opportunities in the past; however, only three continuing-education “products” [17] are currently available. According to ASHA, it is rare for members to access tinnitus-related opportunities; fewer than ten individuals have completed any tinnitus-related work in the past year. The AudiogyOnline [18] continuing-education service offers 52 options, about half of which are linked directly to manufacturers of devices. Most of the course options on AudiologyOnline indicate reviews of the courses that number from 100 to 2170, hence some courses accommodate thousands of viewers across several years. Most of the courses are indicated as “intermediate”; however, presentation modalities are heterogenous, consisting of videos from academic meetings as well as question and answer text offerings. As with ASHA, continuing education is offered online or as recordings; no opportunities for clinical practice are offered and the numbers of consumers are not easily determined [18]. Salus University offers six 1.5-credit modules that cover tinnitus mechanisms, management, and sound tolerance disorders as part of an Advanced Studies Certificate Program. The modules are optional for students completing Salus’ program of study, and are offered entirely online (also offered via AudiologyOnline) and asynchronously in order to maximize availability for international participants. 

One notorious barrier to the provision of tinnitus services remains the professionals’ remuneration for the clinical endeavor. Fee schedules for tinnitus services provided in clinics whose population contains medicare recipients may reimburse basic, albeit often uninformative measures such as pitch and loudness matching. Many professionals conduct tinnitus-related activity as an ancillary service to, and separate corporate entity from, their routine practice. In such cases, patients are required to pay for services out of pocket, therefore the service will be available to a subset of patients who seek help. Tinnitus and sound tolerance issues, for both patients and providers, highlight many of the US healthcare system’s shortcomings; the reimbursement difficulties may represent both a cause and effect of the shortfall of providers and lack of adequate service. It should be noted that, for years, all of the continuing-education and certification training opportunities indicated above were intended to address the shortfall of providers, yet the need remains acute. One additional and widely recognized designator, the Certificate Holder-Tinnitus Management (CH-TM), may support potential providers in a more durable manner and is summarized below. 

Considered in the context of Henry et al.’s [12] findings, it is not likely that such basic barriers could be addressed in any reasonable way with fewer than 16 dedicated hours of course and clinic work throughout a multi-year training program offering a doctoral degree. 

Perhaps extensive clinic placement opportunities could compensate for the dearth of program credit hours, however Henry et al. [12] reported that only 25/32 responder programs indicated externships that offered tinnitus-related opportunities for practice, or “tinnitus-specific mentorship”. Of these, ten indicated that fewer than 25% of the students received such training, while ten indicated that the opportunities were offered to at least half the students; overall, 41% of students received training specific to tinnitus. Tinnitus training outside the University clinic was not consistent, and in nearly half the programs responding, such training was by student request and hence “voluntary”. Yet, when the authors asked program administrators, on a 1–10 scale, whether students were well-prepared to offer services, with 10 signifying “exceptionally well-prepared”, the responses were for the most part positive with regard to making onward referrals (8.8/10), conducting a tinnitus assessment (7.8/10), providing counseling (6.8/10), and performing at least one tinnitus intervention (5.8/10). If the current regrettable state of tinnitus clinical services in the US is an indication of program efficacy, then there are stark differences between the perceptions of program directors regarding their programs and the willingness with which their graduates approach providing tinnitus-related services. No matter how concerned by our deficiencies we are as providers, it may be that our patients are at the same time substantially more concerned. 

### 3.2. Perspectives on Translation and Interprofessional Practice

If students do not receive a critical mass of experience and information supporting tinnitus management during their matriculation through a program, then it may not be reasonable to expect them to embrace the endeavor in their careers. Indeed, the previous statement has relevance for clinical psychology students, who lack access to tinnitus-related instruction, as well as those in hearing science/audiology programs. Increasing the availability and ability of providers would facilitate the translation of scholarship—particularly clinically directed scholarship—into practice at a rate likely greater than at present. Audiology programs, and their clinical partners, could facilitate this process by employing interprofessional models, such as those employed in group intervention settings [19]. In addition to group options, audiologists could recognize self-efficacy training as an intervention strategy routinely employed in past and current practice with regard to fall prevention, hearing aid use, and audiologic rehabilitation. 

The American Board of Audiology introduced in 2018 a certificate program intended to provide practitioners additional training and educational modules associated with clinical practice and the management of tinnitus and sound tolerance disorders [20]. The certificate holder in tinnitus management (CH-TM) completes seven online modules that address the Foundations of Tinnitus Management (tinnitus definitions, management, and business management) and Tinnitus Management Principles in Practice (audiologic evaluation, intervention techniques, and management plans for patients with tinnitus and sound tolerance disorders). Each module requires at least one hour to complete, while some certificate earners use additional time to review notes and study for online evaluations. While the certificate program supports clinical practice in an underserved portion of audiology’s scope of practice, and requires completion of module-specific assessments, it cannot ensure that participants have opportunities to practice in a supervised clinical setting. As of this writing, according to Torryn Brazell, the chief operating officer of the American Tinnitus Association (ATA) [21], 483 professionals have earned the CH-TM through the online offering, 435 of whom are audiologists practicing in the US. Approximately 1000 students graduate audiology programs annually; therefore, fewer than 10% of graduates sought the CH-TM since its inception in 2018. The ATA also reported that their AuD degree-holder membership doubled since the certificate was initially offered. It remains unclear whether the increase in certified providers translates into a meaningful increase in services rendered.

At least three graduates from the author’s AuD program completed the CH-TM during their first few years post graduation. All three graduates acknowledged the value of having the certificate as a designator affirming their clinical competence; however, the graduates also indicated that the certificate program did not extend in a substantive way the class and clinical experiences provided during their matriculation through the AuD program. As an extension of AuD programs of study, the CH-TM may be considered a welcome addition, but should not be considered a substitute for a rigorous full-semester course supported by a rotation in a tinnitus clinic. If the certificate’s completion required two hours/module, then the 14 h total would approximate the typical AuD program time devoted to tinnitus study as reported by Henry et al. [12]. 

### 3.3. Audiologists and Non-Audiologic Management Approaches

Other than medical interventions for cases such as otitis media or otosclerosis, tinnitus sensations persist for most patients regardless of attempts to shut the sound off. At present, the strongest evidence base for tinnitus management is CBT [7,10,16], an intervention that convention would state resides outside audiologists’ scope of practice. Because there are no reliable cures for the ubiquitous subjective tinnitus sensation experienced by nearly 1 billion humans, research and clinical practice in the area of tinnitus often fail to please providers and patients alike. Because patient education and counseling offer non-invasive tools that may, more than other interventions, improve patient self-efficacy and agency, the need for audiologists to convey relevant, accurate, and helpful information to patients is clear. How best to prepare audiologists to address tinnitus and sound tolerance problems in the clinic, given that psychological interventions have the strongest evidence base, remains a challenge.

The observation that bothersome tinnitus, or “tinnitus disorder” as described by DeRidder et al. [22], would emerge when mental health status is “associated with emotional distress, cognitive dysfunction, and/or autonomic arousal, leading to behavioural changes and functional disability” [22] affirms the putative value of interprofessional care opportunities for affected patients. One barrier to patient care specified by Cima et al. [16] is related to the lack of multidisciplinary teams in some European countries, and a similar lack of opportunities exists in many parts of the US. Without support from other professionals, an audiologist might limit tinnitus clinical activity due to their concern regarding extra-auditory contributors to tinnitus disorder. While such trepidation might seem reasonable, it should be pointed out that audiologists routinely fit hearing aids to anxious and irritated patients who resist the devices. The process is far from high-fidelity CBT; however, it is often effective because it relies upon durable tenets of cognitive training, desensitization, and the establishment of realistic expectations for the patient. Audiologists foster patient self-efficacy when providing counseling regarding fall prevention, assistive devices in classrooms, cochlear implant use, and more. Unfortunately, clinicians do not recognize the similarities between the management strategies they already employ and those that could be of benefit to patients with bothersome tinnitus. 

The need for audiology providers to recognize mental health contributions to tinnitus and sound tolerance disorders requires consideration of training components that were viewed in the past as the exclusive purview of clinical psychology training programs. Because tinnitus effects may be exacerbated by co-occurring mental health challenges such as depression or PTSD, audiologists must be prepared to provide onward referrals and appropriate counseling regarding mechanisms shared by tinnitus and mental health status. That tinnitus effects are also compared to those of other conditions such as chronic pain suggests the need to employ patient-centered interventions that support coping and a patient’s ability to talk themselves through challenging situations and environments. The evidence supporting cognitive behavioral approaches to tinnitus management [7,10,16] affirms the value of addressing patient beliefs and of supporting patients’ understanding of tinnitus to the degree that the patient can employ in a reasonable manner lexical items that support an accurate tinnitus narrative. As specified across decades of studies related to the management of traumatic memories, for example, a patient who can employ accurate and comprehensive narratives will more likely be able to manage the effects of traumatic memories and arousal [2,23,24,25,26]. Unfortunately, the lack of preparation offered to audiology students in the majority of AuD programs affects their ability to provide the management strategies and narrative elements required by distressed patients. While it remains unlikely that rank and file audiologists would provide and gain reimbursement for tinnitus-related CBT programs, elements of cognitive training that foster a patient’s tinnitus management may be employed. One such strategy, self-efficacy training, sets its focus on improving patients’ confidence and agency when confronted with a challenging condition that lacks a simple and accessible cure, such as tinnitus or disorders of sound tolerance.

Self-efficacy training is an intervention with a decades-long history of supporting patients in co-existing with chronic conditions for which management, not curing, is the only option. Bandura [27] offered the rationale and evidence to support specific methods by which patients could be counseled and encouraged to employ thought and action in ways that facilitated overcoming barriers and challenges associated with, for example, healthcare needs or a handicapping health condition. Self-efficacy training [27] supports care by enhancing patients’ mastery of challenging activities and experiences, providing vicarious experiences, using verbal persuasion to inform, and improving a patient’s sense of control, thereby influencing physiologic and affective states. 

Mastery experiences resemble interventions that employ “baby steps” as a way to provide the patient evidence that there are elements of the tinnitus experience that may be controlled. A patient may believe that tinnitus interferes with communication; however, the use of elevated test levels to illustrate the influence of amplification followed by hearing aid evaluation may illustrate that communication can be improved even though a tinnitus sensation remains present. In this case, the patient learns that mastering the use of a hearing aid, Bluetooth streaming device, or other sound generator, although not the same thing as mastering tinnitus, facilitates co-existing with an unwanted tinnitus sensation. Other examples of mastery experiences could include the improvement of sleep through the incremental use of sleep hygiene strategies. 

The second self-efficacy objective employs vicarious experiences during which a patient may observe, through other affected individuals, the benefits of counseling, education, device use, or other strategies that reduce distress in others. Group sessions for patients bothered by tinnitus would support this goal as patients would have opportunities to interact with one another, share each other’s successes and learn from each other’s challenges. 

Verbal persuasion is an element of self-efficacy training with which audiologists may feel the most comfortable. Counseling regarding tinnitus mechanisms results from medical assessments that rule out the possibility that tinnitus is a symptom of sinister or terminal illness, and an effective use of communication strategies, for example, may to some degree “persuade” the patient to reassess their prior beliefs and concerns regarding tinnitus and its perceived value. 

Finally, self-efficacy training seeks to increase a patient’s perception of their own ability to control the influences of tinnitus on daily life, including their emotional reaction to the tinnitus. Interprofessional approaches and teams may facilitate improvements in a patient’s coping, and may target elements of tinnitus as it affects emotional and physiologic state. Addressing the patient’s sense of control [28], supports a variety of coping strategies that can minimize the intrusiveness and negative effects of tinnitus. In addition to supporting tinnitus management for patients, the inclusion of self-efficacy principles in audiologic rehabilitation classes could provide a reasonable conduit for students to acquire experience and confidence—self-efficacy of their own—with regard to patient care. Note that audiologists taking on the role of a clinical psychologist is not being advocated, and in no way should an audiologist employing mastery experiences, for example, be thought of as conducting a comprehensive self-efficacy training course. The reader is encouraged to consider whether a subset of elements drawn from interventions such as an 8-week CBT program, or a comprehensive self-efficacy training program, provide value to patients in audiology clinics. If the answer is “yes”, then it is reasonable to provide students an acknowledgement that clinical psychology employs tools that audiologists (and others) may adapt to improve patient outcomes; at the same time, programs would provide the means and opportunities for students to obtain relevant clinical experience. If the answer is “no”, then the practice of audiologic rehabilitation will require recalibration.

A self-efficacy questionnaire intended to assess challenges facing patients with tinnitus was validated [29] and later distinguished the self-efficacy levels across patient groups with and without trauma histories [30]. In those studies, patients with prior military service sought tinnitus-related services at a Veteran’s Medical Center (VAMC) audiology clinic. Patients with PTSD diagnoses, and whose tinnitus was related to traumatic exposures—tinnitus with sudden onset that was traced to specific traumatic events—rated their tinnitus handicap and intrusiveness as more severe than patients without PTSD. The questionnaire identified specific elements of a patient’s daily routine affected by tinnitus, thereby supporting focused management strategies related to, for example, device use, sleep hygiene, and communication. A student project implementing a guided self-efficacy program intended to improve tinnitus management is currently offered in both individual and group settings at our university tinnitus clinic. Results will emerge over the next few months as the program continues. 

### 3.4. Medical Humanities and Tinnitus Education

Education and clinical practice opportunities related to tinnitus may support the patient’s and the clinician’s self-efficacy, and the process by which an individual improves their skills may be informed by the tenets of medical humanities. Kirklin [31] described the Medical Humanities program components at the Royal Free and University College Medical School at the University College of London (UCL). The summary emphasized the merging of diverse curricular elements such as the arts, arts therapy, humanities, and philanthropic activities among others, whose addition to student training supported medical management and intervention delivery. Shapiro et al. [32] asserted that the medical humanities have a “moral function” as the practice should compel students and providers to (re)evaluate their attitudes and actions in order to offer patients accurate, thorough, and relatable information that addresses patients’ prior knowledge, beliefs, and suffering, as well as their perspectives on healing. The use of the humanities in this context is more applied than it would be as an academic endeavor; when linked to medical practice and service delivery, for example, the arts offer a perspective and a language that may resonate with patients in ways that foster adapting to and managing a challenging condition, perhaps one without a simple cure such as tinnitus. Unfortunately, as pointed out by Shapiro et al. [32] and others, “By and large, medical humanities remain an intriguing sideline in the main project of medical education” [32].

Examples of tinnitus considered in a medical humanities context provide counseling elements that can be employed to great effect with patients. Baguley [33] provided a chapter identifying many instances of tinnitus and disorders of sound tolerance appearing in literature and the arts. Such information may be particularly useful for practitioners who participate in interprofessional teams, who employ self-efficacy elements such as verbal persuasion [27], and who recognize elements related to the medical humanities that may provide unusual and helpful perspectives for a patient struggling with their tinnitus experience. Weaving examples from literature and popular culture into a tinnitus counseling session provides the patient a novel view of tinnitus that may facilitate an understanding of its effects and its ubiquity, not just as a modern-day event, but as a durable element of the human condition. 

In a comprehensive book chapter, Stephens placed tinnitus in a historical context [34], and in doing so provided a sort of origin story that can support patient understanding of tinnitus’ ubiquity even during periods of human activity that preceded industrialization. By reviewing the Stephens chapter, the provider may address the frustrations of patients who received conflicting or unhelpful information from other clinicians; the chapter affirms that frustrations and fears were shared by patients more than 2000 years ago. 

Nothing about acquiring such information cures tinnitus; however, the interaction between patient and provider(s) benefits from the broader scope of counseling topics as well as the likelihood that the expansive view of tinnitus and its effects, when incorporating centuries of art and literature, may become relatable to the patient in a manner that reduces some of the tinnitus distress. If tinnitus existed in society prior to loud sound, if people have been bothered by tinnitus for centuries, and if the tinnitus sensation is so common that it can be used as a trope in movies and literature, then the patient may develop an understanding that the sensation is not unique to them despite the observation that they are the only one who hears it. Medical humanities training and implementation seem an ideal a fit for audiology students and practitioners with regard to supporting a patient’s understanding of, and management of, bothersome tinnitus.

## 4. Conclusions

Barriers to the access and effectiveness of tinnitus interventions continue to challenge patients, students, academic programs, audiologists, and otolaryngologists. It is acknowledged that an inadequate number of AuD programs in the US provide for students a substantive set of experiences focused on tinnitus and disorders of sound tolerance in the classroom and clinic. At the same time, while clinical psychologists employ tools of known benefit to patients with bothersome tinnitus, they cannot be counted upon to address on top of their current caseloads a condition with tinnitus’ prevalence. Further, it is more likely that audiologists can gain experience with, and implement on their own, strategies from formal programs such as CBT and self-efficacy training. The audiologist would employ these techniques much as they already do, as elements of rehabilitation intervention. Greater focus than that offered at present on such management strategies, coupled to comprehensive study of tinnitus mechanisms both audiologic and non-audiologic (i.e., psychological) would if nothing else improve student and clinician self-efficacy. The student who is exposed to patients with bothersome tinnitus, and who collaborates on that patient’s management of tinnitus, will be more likely than an unexposed student to work with similar patients in the future. Such opportunities need to be created and fulfilled at a higher rate than at present.

## Data Availability

Not applicable.
